# Supplementary material for: Surface Microbiome Profiling of Dental Elevators Using Third‐Generation Sequencing: Implications for Infection Control in Dental Practice
Source: Microbiologyopen. 2025 Dec 8;14(6):e70178. doi: 10.1002/mbo3.70178 (PMC12685768; doi:10.1002/mbo3.70178)
Supplement: Supplementary file 1 — customer_backup. [file MBO3-14-e70178-s001.zip › customer_backup/customer_backup/otus/flower/treat/treat_flower.pdf]

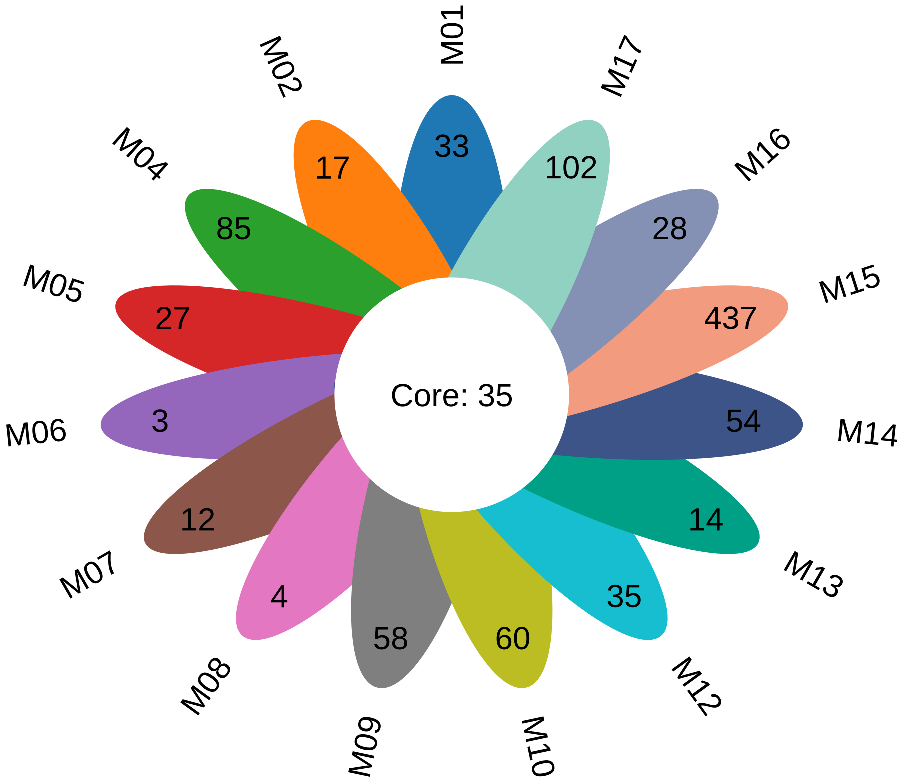
M01
33
M02
17
M17
102
M04
85
M16
28
M05
27
M15
437
M06
3
Core: 35
M14
54
M07
12
M13
14
M08
4
M12
35
M09
58
M10
60
